# Is neighbourhood deprivation in primary school-aged children associated with their mental health and does this association change over 30 months?

**DOI:** 10.1007/s00787-024-02385-y

**Published:** 2024-02-14

**Authors:** Katie Finning, Amy Haeffner, Sohum Patel, Bryony Longdon, Rachel Hayes, Obioha C. Ukoumunne, Tamsin Ford

**Affiliations:** 1 Public Policy Analysis, Office for National Statistics, Newport, UK; 2 Exeter Medical school, University of Exeter, Exeter, UK; 3 School of Clinical Medicine, University of Cambridge, Cambridge, United Kingdom; 4 Department of Health and Community Sciences, Faculty of Health and Life Sciences, College of Medicine and Health, University of Exeter, Exeter, UK; 5 Department of Psychiatry, University of Cambridge, Cambridge, UK

**Keywords:** Child health, Life course epidemiology, Mental health, Social determinants, Deprivation

## Abstract

As both socioeconomic deprivation and the prevalence of childhood mental health difficulties continue to increase, exploring the relationship between them is important to guide policy. We aimed to replicate the finding of a mental health gap that widened with age between those living in the most and least deprived areas among primary school pupils. We used data from 2075 children aged 4–9 years in the South West of England recruited to the STARS (Supporting Teachers and childRen in Schools) trial, which collected teacher- and parent-reported Strength and Difficulties Questionnaire (SDQ) at baseline, 18-month and 30-month follow-up. We fitted multilevel regression models to explore the relationship between Index of Multiple Deprivation (IMD) quintile and SDQ total difficulties score and an algorithm-generated “probable disorder” variable that combined SDQ data from teachers and parents. Teacher- and parent-reported SDQ total difficulties scores indicated worse mental health in children living in more deprived neighbourhoods, which was attenuated by controlling for special educational needs and disabilities but remained significant by parent report, and there was no interaction year group status (age) at baseline. We did not detect an association between probable disorder and IMD although an interaction with time was evident (p = 0.003). Analysis by study wave revealed associations at baseline (odds ratio 1.94, 95% confidence interval 0.97–3.89) and 18 months (1.96, 1.07–3.59) but not 30 months (0.94, 0.54–1.57). These findings augment the existing, highly compelling evidence demonstrating worse mental health in children exposed to socioeconomic deprivation.

**Supplementary Information:**

The online version contains supplementary material available at 10.1007/s00787-024-02385-y.

## Introduction

Mounting evidence suggests that mental health conditions are increasingly prevalent among children and young people in the UK, but also that those with poor mental health face worse subsequent outcomes [1–3]. Existing literature demonstrates a strong association between lower socioeconomic status and poor mental health in children and young people [1, 4–6]. For example, a systematic review of the global literature reported that 52 out of 55 identified studies had at least one marker of deprivation correlating with poor mental health [5]. A population-based cohort study investigating Scottish children starting primary school in 2012 assessed mental health using the Strengths and Difficulties Questionnaire (SDQ) and reported that pupils in the most deprived quintile-based group as classified by their home postcode were nearly twice as likely to have an abnormal SDQ score than their most affluent peers at age four (7.3% vs. 4.1%). Worryingly, this gap widened substantially by age seven (14.7% vs. 3.6% [7]). The prevalence of mental health conditions is known to increase with age [8], but the widening mental health gap between those living in more or less affluent neighbourhoods was a novel, alarming and important finding [7].

The impact of neighbourhood deprivation on mental health may be cumulative due to repeated exposure to adverse childhood experiences and ongoing challenges to development with reduced exposure to factors protecting and improving resilience [9]. The global cost of living crisis and the increasing number of children living in families that face financial, housing or food insecurity makes understanding the relationship between socioeconomic status and mental health particularly important to current health and education policy. For example, the UK has a specific “levelling up” policy [10].

We aimed to explore the mental health of children aged 4–9 years participating in the Supporting Teachers And childRen in Schools (STARS) trial in relation to neighbourhood deprivation, to replicate and expand Marryat and colleagues finding [7]. While the Scottish study reported on children aged 4 and then at age 7 from a single school year, the STARS sample provided a broader age-range, parent as well as teacher report and three waves of data. STARS also allowed us to separate age from time, as data were collected in three overlapping cohorts of 4–9 year olds We also explored the influence of Special educational needs or disabilities (SEND), which refer to impairments in cognitive function, social capabilities, behaviours, or health conditions that impede a child’s ability to learn [11, 12]. The relationship between poor mental health and SEND is likely bidirectional and multifactorial, while SEND is known to be more common among children from socioeconomically deprived groups as well as those with poor mental health [13]. Causality may run either direction; for example, difficulty coping with school resulting from neurodevelopmental disorder, bullying or learning disability may precipitate or maintain mental health conditions, while academic attainment may be compromised by poor mental health [14]. Especially for some children, their mental health condition can be the sole reason for SEND support, such as autism or Attention Deficit Hyperactivity Disorder [2].

Our objectives were to test whether teacher- and parent-reported mental health differed between children living in the most and least deprived neighbourhoods and to examine whether any association differed across age groups or changed over time. Given both Marryat’s [7] and wider findings [1, 4–6, 8], we hypothesised that children living in more deprived neighbourhoods would have worse mental health and that the mental health gap between the most and least socioeconomically deprived would increase with increasing age.

## Methods

This study was a secondary analysis of data collected from STARS; a cluster randomised controlled trial of the Incredible Years® teacher classroom management that ran from 2012 to 2017 in 80 primary schools across the Southwest of England [15]. Each school participated for 3 years, with three overlapping cohorts  recruited in 2012 (*n* = 15 schools), 2013 (*n* = 30), and 2014 (*n* = 35), respectively. Schools were eligible to participate if they had a single year group class of at least 15 pupils aged 4–9 years (Reception to Year 4), who were taught by the same teacher for a minimum of 4 days per week. Schools catering only for children with SEND were excluded, as were schools deemed inadequate by the Office for Standards in Education. Headteachers nominated one teacher to participate in the study, either to act as a control or to attend a teacher classroom management course depending on the trial arm their school was randomised to. All pupils within their class were eligible for inclusion provided their parents did not opt them out of the research and their teacher considered that the parents had sufficient English language comprehension to understand the recruitment information. The study evaluated whether increasing teacher ability to manage challenging classrooms improved child well-being, behaviour and academic attainment. Trial data collection occurred before randomisation (baseline) and at 9, 18 and 30 month follow-ups. The STARS trial had ethical approval from the Peninsula College of Medicine and Dentistry Research Ethics Committee (12/03/141).

### Measures

#### Socio-economic status

**Fig. 1:**
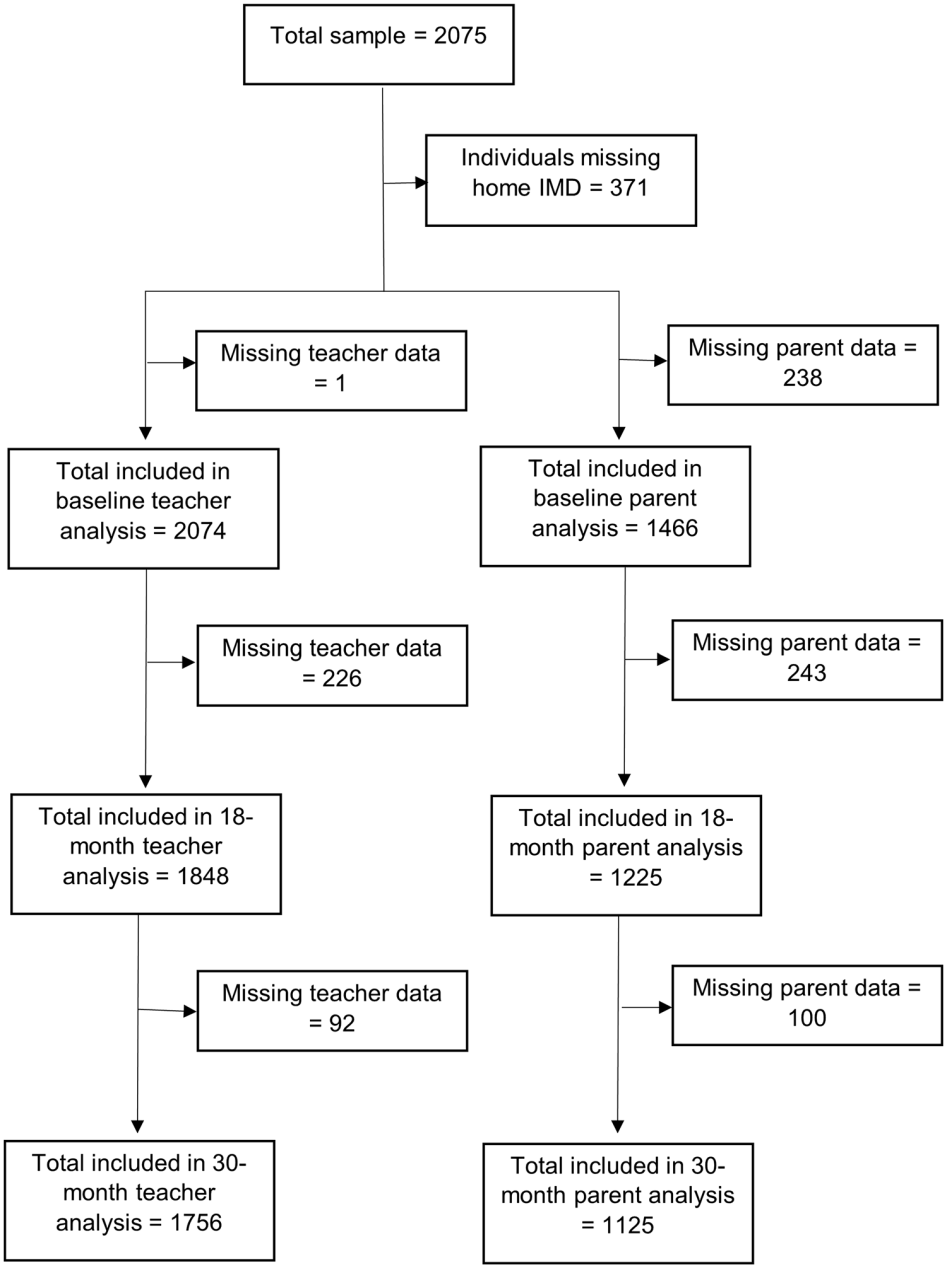
Data from parents and teachers available to the analysis throughout the study

Socio-economic status was quantified using the Index of Multiple Deprivation (IMD) obtained by linking the postcode data from the child’s home address, where available from parental report (see Fig. 1). IMD is a national index of relative deprivation in small areas of England [16], determined by ranking area-level scores on 37 indicators grouped into seven domains that reflect different aspects of deprivation: (i) income, (ii) employment, (iii) education, skills and training, (iv) health deprivation and disability, (v) crime, (vi) barriers to housing and services and (vii) living environment [17]. We categorised the neighbourhood deprivation that children experienced, based on the IMD, into five quintile-based groups determined using cut points from national data; from most deprived (1) to least deprived (5—reference category). IMD was only classified at baseline, although we did follow-up children who moved school during the study where possible.

#### Mental health

Teachers and parents completed the Strengths and Difficulties Questionnaire (SDQ) at baseline, 9, 18 and 30 months. The SDQ is a 25-item questionnaire used to evaluate common mental health problems among 4–16 year olds, with parallel versions for teachers and parents that contain identical items. Responses to each item are on a three-point Likert scale; not true (0), somewhat true (1) and certainly true (2) for difficulties, or reversed for strengths, so that a high score indicates greater difficulty. The SDQ total difficulties score is calculated as the sum of the scores for 20 of these items, which address emotional problems behavioural difficulties, peer relationships and attention / concentration. The possible score ranges from 0 to 40. In a national sample of school-aged children, the Cronbach’s alpha (*α*) value for internal consistency was 0.87 and the test–retest stability over 4–6 months as quantified by the Pearson correlation coefficient (*r*) was 0.80 for the teacher-reported total difficulties score [18]. Similar results were obtained for parent-reported total difficulties (*α* = 0.82; *r* = 0.72). The impact supplement asks about impairment to classroom learning, peer relationships (both parents and teachers), family life and leisure activities (parents only).

A computerised algorithm can be used to predict the likelihood of mental health conditions by combining scores on the emotions, behaviour and attention concentration scores with evidence of impairment using data from all available informants; in the current analysis this was teachers and parents [18, 19]. The algorithm generates ratings (“unlikely” symptoms < 3 for behaviour / emotions or < 5 for attention and impact score = 0; “possible” or “probable” symptoms score > 95th centile and Impact score > 2). We created a binary variable for children to indicate if any disorder was “unlikely/possible” versus a “probable”, using the conventional classification [20].

#### Demographic and background characteristics

Data on the following were provided by parents at baseline: child gender (male or female), ethnicity (categorised from free text and aggregated into White versus Other due to a small number of children from ethnic minority groups), free school meal eligibility, SEND status, the highest qualification obtained by the child’s parent, whether English was spoken as an additional language, number of children in the household and whether the child lived in rented or owned accommodation. In England, a child is deemed to have SEND if their ability to function in the school environment is impaired by a cognitive, learning, health or other difficulty to the extent that additional support is required.

#### Analysis

Analyses were undertaken using Stata/SE 16.1 [21]. Baseline characteristics of the children were summarised using means and standard deviations for continuous variables, and numbers and percentages for categorical variables. Parental report of SDQ was missing for 609, 850 and 950 participants in the initial, 18-month follow-up and 30-month follow-up, respectively. Mean teacher-reported SDQ score was compared for those missing and not missing parent data to identify difference—if any—dependent upon parental engagement with the trial, as reported in Supplementary Fig. 1. Parental non-response also resulted in missing sociodemographic data on ethnicity, highest household qualification and eligibility for free school meals. We compared responses to STARS to national survey data [23] for children of the same age to assess the generalisability of our sample.

We studied three outcome variables; namely teacher and parent report SDQ total difficulties scores and probable disorder as derived by the algorithm and explored associations between socioeconomic status (based on IMD quintile-based groups) and outcome variables in: (a) crude analyses adjusted for study wave only; (b) analyses adjusted for study wave and the potential confounders (child’s age at baseline, gender, ethnicity and trial arm status); (c) a sensitivity analyses adjusted for study wave, the potential confounders and SEND status. Our primary analysis was analysis was (b) and excluded SEND to match Marryat's work [7].

Each continuous outcome (teacher-reported and parent-reported SDQ total difficulties scores) observations across baseline, 18 months and 30 months were included in repeated measures analyses, fitting mixed effects (“multilevel”) regression models to allow for the correlation between observations from the same pupil and correlation between pupils from the same school—i.e., a three-level model was fitted with repeated observations nested within pupils nested within schools. Mean differences were reported for these analyses. The binary outcome (probable mental disorder versus unlikely/possible) was analysed using logistic regression with information sandwich (“robust”) estimates of standard error to allow for the correlation between pupils from the same school. We tried to fit three-level mixed effects logistic regression models, but these sometimes failed to converge and so we used a simpler analysis approach that only allows for correlation between pupils in the same school. Examination of the analyses that did converge for a three-level mixed model versus our simpler approach indicated that ignoring the correlation of repeated observations in the same pupil made little difference to the findings. Odds ratios were reported for these analyses. All models included study cohort as a fixed effect. The least deprived IMD (quintile 5) was specified as the reference group for the analyses. We present mean differences and odds ratios, which represent the pooled estimates of the relationships between socioeconomic status (IMD quintile group) and mental health outcomes across these three study cohorts. Because analyses of the intervention in the STARS trial indicated a small but statistically significant effect of teacher classroom management (TCM) at 9 months follow-up, the analyses in this paper used data collected at only the baseline, 18-month and 30-month waves [15]. We excluded the 9 month follow-up data from the current analysis given the difference among children in the intervention arm of the STARS trial on teacher but not parent-reported mental health [mean difference in SDQ total difficulties score = 1.0 (95% CI 0.1–1.9; (*p* = 0.03)] detected at 9 months but not at subsequent follow-ups. However, children with a teacher-reported total difficulties score that was greater than 12, the clinical cutpoint, had lower teacher report total SDQ scores (so better mental health) across all three follow-ups, which suggests a differential response to the Incredible Years intervention by baseline mental health and is why we adjusted for trial arm status [15].

Tests of interaction were undertaken between IMD quintile-based group and year group at baseline to examine whether the associations between neighbourhood deprivation and the three outcome variables were different for across year groups, and between IMD quintile-based group and study wave (baseline versus 18 months versus 30 months) to examine whether the associations changed as the children got older.

To handle missing data in both our predictor and outcome variables, we used multiple imputation on the assumption that data were missing at random (i.e., that missingness was accounted for by other variables within the dataset) [22]). STARS study recruited 2075 pupils (see Fig. 1) but 371 children (18%) lacked a parent report so a residential IMD could not be classified. Furthermore, data were missing about individual children due to non-response at 18 months and 30 months follow-up for 227 (11%) and 319 (15%) of teacher-reported SDQ and for 850 (41%) and 950 (46%) of parent-reported SDQs. Parental non-response also resulted in missing sociodemographic data on ethnicity, highest household qualification and eligibility for free school meals. Children who were missing parent-reported data across all time-points and at each individual timepoint had consistently higher mean teacher-reported SDQ total difficulties scores at each wave (at baseline, mean for those missing data = 6.2, those not = 9.5; at 18 months, missing data = 5.8, those not = 8; at 30 months, missing data = 5.6, those not = 8. *p* < 0.05 for all as tested by paired *t* tests) (See Supplementary Fig. 1).

Fifty imputed datasets were created using the chained equations method, with Stata’s *mi impute chained* command. Variables included in the imputation model included the exposure (IMD quintile-based group), mental health outcomes and confounding variables described above. The following auxiliary variables were also included: highest level of education attained by the parents, whether English was spoken as an additional language, eligibility for free school meals, number of children in the household, school IMD quintile, whether the child lived in rented or owned accommodation and baseline and follow-up SDQ impact scores reported by teachers and parents. In addition to our main analyses using multiply imputed datasets, we performed sensitivity analysis using complete cases. Results from complete case analyses (*n* = 1102) were similar to those obtained in the analyses of imputed data.

## Results

Slightly more than half the sample were boys, and as is typical of the population in the South-West of England, just under 5% were from an ethnic minority or spoke English as a second language (see Table 1). The sample was evenly divided between educational stage Key Stage 1 (Reception, Years 1 and 2) and Key Stage 2 (Years 3, 4), while 20% of children were living in the most deprived quintile, 8% were receiving free school meals and 21% had SEND according to school report. Parent-reported mean SDQ total difficulties score was similar (mean = 7.8 standard deviation (SD) = 6.0) to the equivalent reports in the English Mental Health Survey for Children and Young People in 2017 for 4–10-year-olds (Mean = 7.9, SD = 5.9, *t* = 1.4645; *p* = 0.14), as was the proportion with probable disorder (9.6% STARS versus 9.9% 2017 national sample) [23]. In contrast, STARS teachers reported a statistically higher level of difficulty (mean 6.7, SD = 5.9) than the English population as a whole (mean = 3.0, SD = 5.5, (*t* = − 39.81; *p* = 0.0000).

**Table 1:** Baseline sample characteristics (*n* = 2075 pupils in STARS^a^)

Characteristics	
Age: mean (SD)	6.26 (1.3)
Year group:* n *(%)	
Reception	270 (13.0)
Year 1	368 (17.7)
Year 2	410 (19.8)
Year 3	609 (29.3)
Year 4	418 (20.1)
Sex: *n* (%)	
Male	1101 (53.1)
Female	974 (46.9)
Ethnicity: *n* (%)	
White	1352 (95.1)
Other	70 (4.9)
Household IMD Quintiles: *n* (%)	
1 (Most deprived)	344 (20.2)
2	402 (23.6)
3	415 (24.4)
4	342 (20.1)
5 (Least deprived)	201 (11.8)
Parental qualifications: *n* (%)	
None	75 (5.0)
GCSE/A-level	754 (50.5)
Degree or higher	663 (44.4)
English language: *n* (%)	
English not additional language	1450 (95.7)
English is an additional language	66 (4.4)
SEND: *n* (%)	
No SEND	1635 (78.8)
SEND	440 (21.2)
Free school meals eligibility: *n* (%)	
No	963 (59.2)
Yes	134 (8.2)
Don’t know	51 (3.1)
Not applicable	479 (29.4)
Number of children in household: mean (SD)	2.3 (1.0)
Parent SDQ total difficulties score: mean (SD)	7.8 (6.0)
Teacher SDQ total difficulties score: mean (SD)	6.7 (5.9)
Psychiatric disorder: *n* (%)	
Unlikely/possible	1875 (90.4)
Probable	199 (9.6)

Sample size is 2075 apart from ethnicity (*n*= 1422), household IMD (*n* = 1704), parental qualifications (*n *= 1492), English language (*n* = 1516), free school meals eligibility (*n*= 1627), number of children in household (*n *=1517), parent SDQ total difficulties score (*n*= 1466), teacher SDQ total difficulties score (*n* = 2074), and psychiatric disorder (*n* = 2074).

*GCSE* General Certificate of Secondary Education, *IMD* Index of Multiple Deprivation, *SD* Standard Deviation, *SDQ* Strengths and Difficulties Questionnaire, *SEND* Special Educational Needs and Disability.

^a^*n* max.

There was evidence of worse mental health among children living in the more deprived neighbourhoods, based on the teacher- and parent-reported SDQ total difficulties score (see Table 2). The relationship was stronger and more consistent according to parent report (evident across quintiles 1, 2 and 3) than teacher report, where it was evident only for the most deprived quintile (1). We did not detect an association with probable disorder when tested as a main effect. With further adjustment for SEND, the association between mental health and IMD was attenuated for all outcomes, and only remained established for the parent-reported SDQ score (*p* < 0.001).

**Table 2:** Relationship between IMD and mental health estimated across all time-points (*n* = 2075 pupils)

	IMD quintile-based group								*p* value
	1 (Most deprived)		2		3		4		
	Effect estimate	95% CI	Effect estimate	95% CI	Effect estimate	95% CI	Effect estimate	95% CI	
Unadjusted	1.8	0.5 to 3.0	1.1	− 0.1 to 2.3	0.6	− 0.5 to 1.8	0.2	− 1.1 to 1.4	0.01
Adjusted^a^	1.7	0.5 to 2.9	1.1	− 0.05 to 2.2	0.5	− 0.6 to 1.6	− 0.004	− 1.2 to 1.2	0.006
Adjusted plus SEND	1.0	− 0.2 to 2.2	0.7	− 0.4 to 1.8	0.5	− 0.5 to 1.6	0.2	− 0.9 to 1.3	0.37
Unadjusted	2.8	1.8 to 3.9	1.8	0.8 to 2.9	1.1	0.1 to 2.2	0.1	− 0.9 to 1.2	< 0.001
Adjusted^a^	2.7	1.7 to 3.8	1.8	0.8 to 2.8	1.0	0.02 to 2.0	0.02	− 1.0 to 1.1	< 0.001
Adjusted plus SEND	2.1	1.1 to 3.2	1.5	0.5 to 2.5	1.0	0.1 to 2.0	0.1	− 0.9 to 1.1	< 0.001
Unadjusted	1.39	0.90 to 2.16	1.08	0.69 to 1.70	1.02	0.67 to 1.56	0.80	0.49 to 1.30	0.09
Adjusted^a^	1.38	0.88 to 2.16	1.06	0.67 to 1.68	0.98	0.63 to 1.52	0.76	0.47 to 1.25	0.06
Adjusted plus SEND	1.11	0.69 to 1.79	0.91	0.56 to 1.49	0.96	0.61 to 1.53	0.82	0.49 to 1.35	0.64

Comparator group is Quintile 5 (least deprived). Effect estimates compare quintile-based IMD categories to the least deprived (reference) category; these are regression coefficients (mean differences) where the outcome is SDQ score (continuous), and odds ratios where the outcome is probable psychiatric disorder (binary). Adjusted models controlled for age at recruitment, gender, ethnicity and trial arm status; study cohort a fixed effect. Effect estimates are reported to two or more significant figures where appropriate to aid understanding.

^a^Primary analysis is without adjustment for SEND. Please note the main analysis is the adjusted analysis that excludes SEND.

P values indicate the strength of evidence of a relationship between the outcomes and IMD quintile.

Tests of interaction indicated little evidence that the relationship between IMD quintile and SDQ score differs according to year group (age) at recruitment; for teacher- and parent-reported SDQ total difficulties score, the *p* values for the tests of interaction were not associated at the 5% significance level. In contrast, the test of interaction considering probable psychiatric disorder indicated a robust difference in the association by timepoint. When analysed by data point (see Table 3); children in the most deprived quintile (1) had a higher prevalence than their less deprived counterparts to have a probable disorder at baseline (marginal association) and 18 months, but not at 30 months.

**Table 3:** Association between IMD and probable psychiatric disorder status—subgroup analyses by timepoint (*n* = 2075 pupils)

	IMD quintile-based group							
	1 (Most deprived)		2		3		4	
	Odds ratio	95% CI	Odds ratio	95% CI	Odds ratio	95% CI	Odds ratio	95% CI
Baseline	1.94	0.97 to 3.89	1.81	0.91 to 3.59	1.36	0.68 to 2.73	1.21	0.56 to 2.60
18 months	1.96	1.07 to 3.59	1.25	0.67 to 2.30	1.19	0.66 to 2.15	0.58	0.27 to 1.22
30 months	0.94	0.57 to 1.54	0.69	0.43 to 1.11	0.68	0.43 to 1.08	0.67	0.41 to 1.09

Comparator group is Quintile 5 (least deprived). Models are adjusted for age at recruitment, gender, ethnicity and trial arm status; study cohort a fixed effect. Please note the main analysis is the adjusted analysis that excludes SEND

*p* value for test of interaction between timepoint and IMD quintile-based group = 0.003

## Discussion

We examined the relationships between teacher and parent reports of primary school children’s mental health and neighbourhood area deprivation over time. We found an association between living in a socioeconomically deprived area and SDQ total difficulties scores from both informants across the three time-points. We found no such association for probable disorder, which we explore further below. The relationship between mental health and SEND is complex [10]; neurodevelopmental conditions in themselves can require support for children to cope with school, while other types of SEND can precipitate mental health conditions and both SEND and mental health conditions are commoner among those of lower economic status. We, therefore, included a sensitivity analysis to explore the effect of SEND, which attenuated the association with all outcomes although the relationship of IMD and mental health remained related (*p* < 0.001) according to parent report. Our main analysis excluded SEND as the study we aimed to replicate did not include it, and because of the complexity of variable that for different children may be a confounder, a mediator or a risk factor.

We failed to replicate the widening of the disparity in mental health by socioeconomic deprivation with age that was reported by Marryat et al. [7]. Time related findings may result from an age effect (mental health deteriorates with age); a cohort effect (related to the particular population of children starting school) or a period effect (relating to children more widely in that context and that date, usually due to particular socio-political events). The latter seems unlikely as data were collected in similar time periods, and although the studies were in different countries with some differences in education and health policy, they are both part of the UK. There are also important methodological differences between their study and ours. This Scottish study was both larger (3166 participants) and all the children shared the same year group and age; the authors dichotomised the SDQ scores; and did not formally test for an interaction with time, although the graphical illustration reported is compelling. In contrast, STARS recruited 2075 children divided across five school year groups, so had less statistical power to detect differences in the relationships across a wider range of ages.

Dimensional measures provide greater statistical power to detect an association than binary variables, which may explain our failure to detect of an association for probable disorder. Our findings might also result from an affect that does not differentially influence those with poorest mental health but operates across the distribution of scores. The SDQ diagnostic algorithm most accurately predicts disorder when combining parents’ and teachers’ reports for this age group, as we did, compared to teacher or parent only report [18–20]. Multi-informant assessment is more accurate than single informant as is the use of impairment as well as symptoms, hence our inclusion of this measure [24]. However, the algorithm’s performance as a diagnostic test is moderate [24], and although better than a cutpoint in symptoms alone, misclassification may also explain our failure to detect an association with caseness. We used the conventional classification (probable versus, unlikely / possible) as it is likely that shifting the cutpoint to probable / possible versus unlikely would serve to increase misclassification and to move the measure further away from clinical diagnoses.

The interaction of probable disorder with time may be a chance finding, particularly lack a similar interaction with parent and teacher SDQ total difficulties scores, for which there was a clear main effect. There is no theoretical reason why those living in deprived areas should be at higher probability of psychiatric disorder at baseline and 18 months but not 30 months.

Interestingly, our findings suggest that a stronger association between parent-reported mental health and IMD. Teacher and parental agreement on mental health measures is surprisingly low [25], although Marryat and colleagues relied on teacher report. For some children, there will be a true difference in how children function at home and at school, as many children, particularly those with anxiety or neurodevelopmental disorders, contain or mask their difficulties in school and then decompensate at home. Parents with a lower socioeconomic status are more likely to have poorer mental health themselves, and their mental state may influence how they score their children on the SDQ [26]. Evidence supports such a reporting bias but direct observation also suggests their children also have more difficulties, and there is no reason for parental reporting bias among respondents to only manifest at baseline and 18 months [27]. The percentage of individuals meeting multi-informant standardized diagnostic assessment for an impairing DSM IVR diagnosis whose algorithm-derived variable indicated probable disorder was 50%, compared to 11% for possible and 2% for unlikely [20]. There is a risk that the relationship between deprivation and diagnosable psychiatric disorder might have been obscured by misclassification, although the use of impairment and multiple informants improves on the use of a simple cutpoint as applied by Marryat and colleagues. While our parent reports reflect national survey data, our teacher reports indicated greater psychopathology among the STARS participants, which probably reflects methodological differences. The national survey recruited parents and requested access to teachers, but not all families accepted and not all teachers responded. Teacher information in the national survey was less likely to be available for children reaching diagnostic criteria for mental health conditions and of lower socioeconomic status.

The children participating in our study experienced a very different environment than the Scottish study, albeit at a similar period (2012–2015 Marryat vs. 2012–2017 STARS). STARS recruited schools from mainly rural and semi-rural areas in South-West England compared to a large Scottish city. Although both samples were living in countries within the UK, England and Scotland differ substantially in the provision of education and social care. Notably, the regions from which the data were collected show similar levels of wealth inequality as measured by the Gini coefficient (59% South-West England and 62% Scotland, respectively) [28]. Some evidence suggests that access to rural areas may be beneficial to mental health, which perhaps might mitigate the impact of deprivation [29–32]. Further research could explore whether this is the case among young children.

Others argue strongly about the deleterious impact of the widening gap between the most and least privileged of British society [33] and given that our findings add to the robust literature that the mental health of children living in poverty seems generally poorer, there is an urgent necessity to support vulnerable children and families, as suggested by Fazel [34]. The Covid-19 pandemic has amplified the significant deterioration in young people’s mental health seen in the first 2 decades of this century [23, 35], with repeated reports that children living in deprived areas or lower socioeconomic status families were more likely to experience poor mental health than their peers who were not facing such challenges [36–38]. The school closures and disruption to education resulting from COVID-19 is predicted to increase the gap in education outcomes between children from poorer and affluent families [11]. Children with pre-existing mental health conditions and SEND were particularly likely to experience poor mental health during the pandemic [37]. Furthermore, there is increasing evidence of the syndemic impact of COVID-19 and resulting restrictions, with vulnerable groups facing multiple challenges that did not affect other sectors of society [39–41]. For example, in a study that compared mental health trajectories during 2020, adults from ethnic minorities, people living in more socioeconomically deprived circumstances and parents with young children, were more likely than their White, more affluent and childless peers to experience deteriorating or consistently poor mental health [42]. We should be particularly concerned that parents of young children emerged as a novel high-risk group [42], given the strong bidirectional relationship of parental and child mental health [43]. This association unique to parent–child connections may offer explanation towards the more pronounced correlation between deprivation and parent-reported SDQ total difficulties score as opposed to those scores reported by teachers. Parents raising children in areas experiencing more deprivation may not only struggle more with mental health difficulties themselves but also be more acutely aware of these difficulties in their children [42, 43].

We need a concerted cross-sector policy to support children and families, which should comprise universal, targeted and indicated measures with a focus that is broader than just mental health. We found that adjusting for SEND attenuated the relationship between deprivation and mental health according to all three outcomes, although the association remained intact (*p* < 0.001) according to parental report. It is essential that children who struggle at school are adequately supported. Prevention should support parenting, provide high-quality affordable childcare and ensure children are adequately fed and housed. Either universal or targeted screening, if linked to effective intervention, might reduce the developmental price of untreated mental health difficulties. Children might also be more likely to respond to intervention if they access support before their difficulties become entrenched [44]. A study of schools in socially deprived areas found that 82% of parents agreed that regular mental health screening in primary schools would be beneficial for students [45], although subsequent access to support needs careful consideration. Mental health services for children are already struggling to deal with referrals, with evidence that many with clinically impairing difficulties struggle to access care [37, 46, 47]. Therefore, we need to ensure that scarce specialist resources are used cost-effectively and to upskill and support all professionals who work with children and young people to provide effective help for milder and transient problems and to identify those who need more active intervention. The aspiration to significantly expand access to high-quality community mental health services to meet the needs of 35% of young people in need by 2021 should aim for complete coverage and risks being swallowed by increasing demand [48, 49]. A focus on economically deprived areas might help target resources where they are most needed and may reduce both individual burden on sufferers and systematic burden on health, social care and education services [50].

There are several strengths to our partial replication, which include the use of a moderately large sample, followed prospectively and the use of a widely used validated outcome measure. We extend Marryat’s work by the inclusion of parents as well as teacher SDQs and the algorithm-generated “probable disorder”. Inevitably, there are constraints with missing parental reports (and, therefore, demographic information and home—IMD), which increased over time. It seems likely that a high proportion of parents who chose not to respond or dropped out of the study were from lower socioeconomic backgrounds [51]. The schools recruited were representative of schools in the South West of England, but we excluded from the trial schools deemed to be unsatisfactory on inspection, which are likely to serve particularly deprived catchment areas [15]. Similarly, the South West of England is an area of low ethnic diversity, so our findings cannot be generalised to more ethnically mixed neighbourhoods.

We chose IMD as our measure of deprivation given our aim to replicate Marryat’s work, which used the Glasgow IMD [7]. IMD was also preferred as it considers a number of markers in its calculation, making it an effective marker of area-level deprivation [16, 17]. IMD has been praised for its use as a comprehensive indicator of area-level deprivation, used by governmental bodies and directing attention to areas in need. However, challenges include those ascribed to each of the 37 included measures, and  the IMD assumes that of each of these measures are experienced similarly by the all individuals in each area [52]. Our work could not explore indictors of individual level socioeconomic status such as eligibility for free school meals and the highest level of parental education, which we did use to impute data, because these characteristics are collinear with our outcome measure.

More generally, residual confounding may explain the finding of an interaction between IMD, probable disorder and timepoint. Further research should seek to replicate our work exploring the mental health gap between children by neighbourhood deprivation in larger datasets and over a wider age-range.

## Conclusions

We demonstrated a relationship over time between living in a deprived neighbourhood and parental and teacher reports of poor mental health among children aged 4-9 years of age that persisted but did not widen over the next 30 months. Given the impact of the pandemic on the mental health of children and parents, plus the increasing number of families currently living in deprived circumstances, our work adds to the large body of work that emphasises the need to better understand and more importantly to address the negative outcomes of deprivation as a matter of urgency.

## Supplementary Information

Below is the link to the electronic supplementary material.

Supplementary file1 (PNG 56 KB)

## Data Availability

Data are available via request from TF.

## Code availability

All code for the analysis presented in this paper is available via GitHub: https://gist.github.com/katiefinning1/0e61c71eb74c3032805adad1477149ca
