# Supplementary figures and images for: Is neighbourhood deprivation in primary school-aged children associated with their mental health and does this association change over 30 months?
Source: Eur Child Adolesc Psychiatry. 2024 Feb 14;33(9):3111–21. doi: 10.1007/s00787-024-02385-y (PMC11424695; doi:10.1007/s00787-024-02385-y)

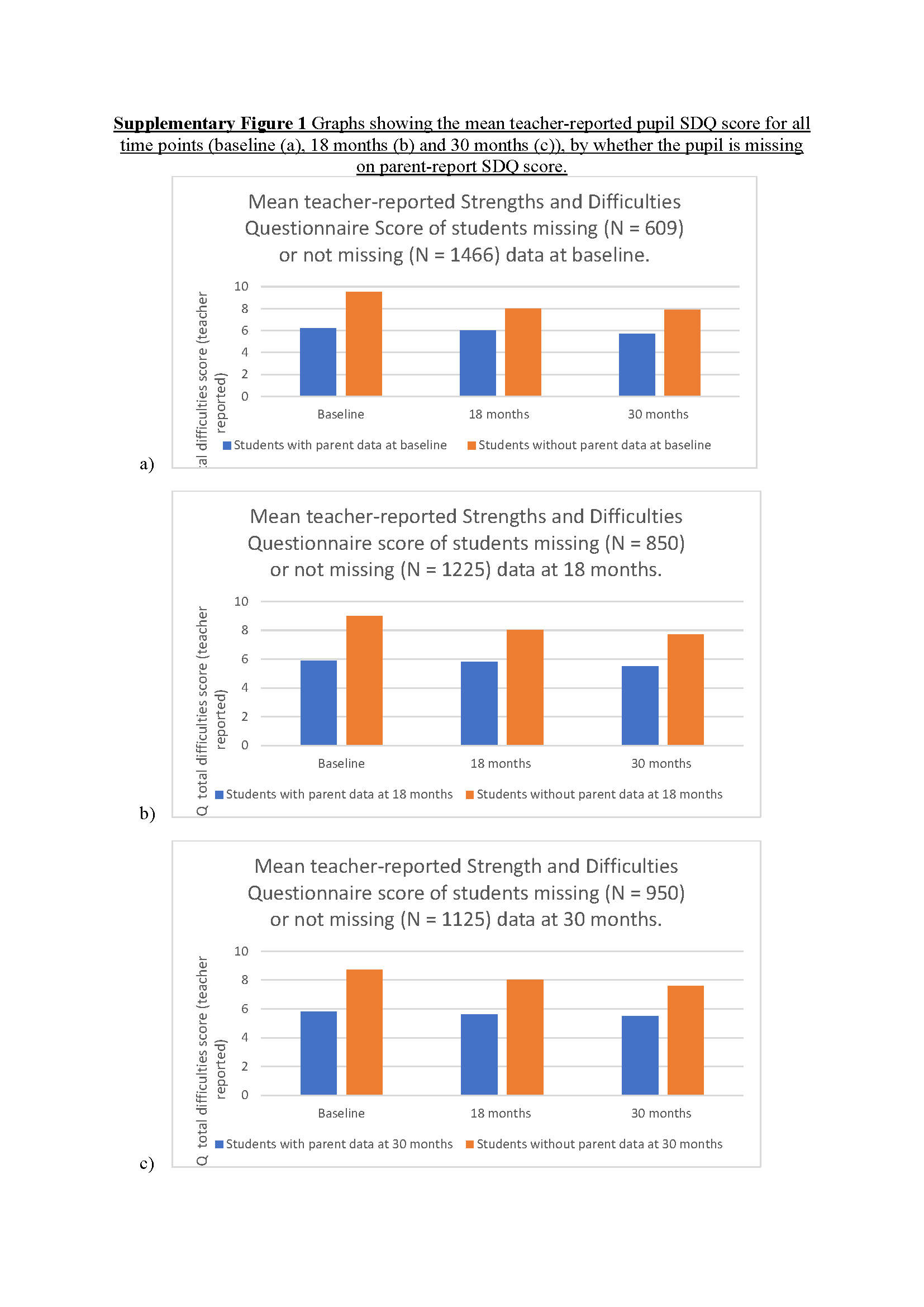

Supplement: Supplementary file 1 — Supplementary file1 (PNG 56 KB) [file 787_2024_2385_MOESM1_ESM.png]
